# Macrophages in Osteosarcoma Immune Microenvironment: Implications for Immunotherapy

**DOI:** 10.3389/fonc.2020.586580

**Published:** 2020-12-10

**Authors:** Zhong-Wei Luo, Pan-Pan Liu, Zhen-Xing Wang, Chun-Yuan Chen, Hui Xie

**Affiliations:** ^1^ Department of Orthopedics, Xiangya Hospital, Central South University, Changsha, China; ^2^ Movement System Injury and Repair Research Center, Xiangya Hospital, Central South University, Changsha, China; ^3^ The Department of Dermatology, Xiangya Hospital, Central South University, Changsha, China; ^4^ Department of Sports Medicine, Xiangya Hospital, Central South University, Changsha, China; ^5^ Hunan Key Laboratory of Organ Injury, Aging and Regenerative Medicine, Changsha, China; ^6^ Hunan Key Laboratory of Bone Joint Degeneration and Injury, Changsha, China; ^7^ National Clinical Research Center for Geriatric Disorders, Xiangya Hospital, Central South University, Changsha, China

**Keywords:** osteosarcoma, tumor microenvironment, macrophages, tumor-associated macrophages (TAMs), immunotherapy

## Abstract

Osteosarcoma is a malignant primary bone tumor commonly occurring in children and adolescents. The treatment of local osteosarcoma is mainly based on surgical resection and chemotherapy, whereas the improvement of overall survival remains stagnant, especially in recurrent or metastatic cases. Tumor microenvironment (TME) is closely related to the occurrence and development of tumors, and macrophages are among the most abundant immune cells in the TME. Due to their vital roles in tumor progression, macrophages have gained increasing attention as the new target of tumor immunotherapy. In this review, we present a brief overview of macrophages in the TME and highlight the clinical significance of macrophages and their roles in the initiation and progression of osteosarcoma. Finally, we summarize the therapeutic approaches targeting macrophage, which represent a promising strategy in osteosarcoma therapies.

## Introduction

Osteosarcoma is one of the most common aggressive malignancies of bone tumors in children and adolescents (1, 2). With improved surgical techniques and neoadjuvant chemotherapy, limb-salvage surgery combined with systemic chemotherapy has been a better option than simply amputation. These multidisciplinary combination treatments have increased 5-year survival to 60–70% in non-metastatic patients with osteosarcoma (3). Despite great success in osteosarcoma management, improvements in survival rates in the last decade were limited (4). Moreover, tumor metastasis or recurrence of patients have consistently shown poorer outcomes and remain unresolved (5). As such, new therapeutic strategies are urgently needed.

Recently attention has been paid to the tumor microenvironment (TME), which plays a crucial role in cancer initiation and progression (6, 7). TME is constituted by tumor cells, fibroblasts, endothelial cells, immune cells, various signaling molecules, and extracellular matrix (8). Due to the complexity and heterogeneity of cells, TME has diverse effects during different stages of cancer progression and metastasis (9, 10). Tumor-associated macrophages (TAMs), as the primary immune cells in the TME, have been identified as a prognostic marker and a new target in tumor immunotherapy (11). A thorough and comprehensive understanding of macrophages may provide new insights and potential therapeutic approaches for osteosarcoma (12–14). Therefore, we briefly introduce the origin, polarization, and regulation of macrophages. Then we focus on the relationship between the polarization status and prognosis of macrophages in osteosarcoma and elaborate on the mechanisms of macrophages in the development and metastasis of osteosarcoma. Finally, targeting macrophages therapy in osteosarcoma is also discussed.

## Overview of Macrophages

Macrophages are generally thought to be developed from the hematopoietic stem cells (HSCs) and derived from the myeloid-monocytic lineage. They are initially recruited from the peripheral blood to eliminate harmful pathogens, infection and inflammation (15, 16). Nevertheless, in recent years, the increasing evidence indicates that tissue-resident macrophages develop from embryos before the appearance of HSCs and maintain self-renewal proliferation (17, 18). Thus the origin of macrophages can be simply divided into two categories: one is tissue-resident macrophages mainly derived from the yolk sac and fetal liver; another is originated from bone marrow-derived blood monocytes (19).

Although the content may significantly vary in different tumors, tumor-associated macrophages (TAMs) are primary immune cells present in the tumor microenvironment. Both circulating monocytes and tissue-resident macrophages contribute to the accumulation of TAMs. The secreted chemokines from tumor cells and stromal cells, such as macrophage colony-stimulating factor (M-CSF) and C-C motif ligand 2 (CCL-2), can induce and recruit monocytes to the tumor microenvironment (20, 21). Notably, it has been found that TAMs were recruited by interleukin-34 (IL-34) released from osteosarcoma cells and infiltrated massively into osteosarcoma tissues (22). These monocytes can differentiate into macrophages under the stimulation of local signal molecules (19).

Macrophages are plastic to multiple signals under the specific TME. The activated macrophages, distinct from tissue-resident macrophages, develop specific phenotypes that show different polarization states and functions (20, 23). Traditionally, a dichotomous spectrum including M1 and M2 phenotypes represented two polarized terminals of the broad range of macrophage activation: classically activated macrophages (M1), stimulated by interferon-*γ*, lipopolysaccharide (LPS) and Toll-like receptor (TLR); and alternatively activated macrophages (M2), activated by cytokines such as IL-4 and IL-13 and other signal molecules (17, 18). Nevertheless, it is also worth noting that macrophages are a heterogeneous population of myeloid cells and have been recognized as a complex spectrum of activation states, represented by a mixed or intermediate phenotype expressing both M1 and M2 markers albeit to a different extent (20, 21). This spectrum model of macrophages suggests a continuum of functional status and can better generalize the real state of macrophage activation in the microenvironment (24–26). Furthermore, due to the lack of specificity of marker expression, the classification based on polarization status *via* a single M1/M2 marker may simplify the complexity of macrophages. For instance, Arginase-1 can be upregulated upon M1 (LPS) or M2 (IL-4) stimulation (17).

Similarly, due to the plasticity and heterogeneity, TAMs are characterized by pro- or anti-tumor activity according to the tumor types and their interactions in the TME (21, 27). TAMs showing M1-like features have the potential to kill tumor cells and enhance the immune response. However, TAMs, generally exhibiting an M2-like immunosuppressive phenotype in most tumors, tend to promote angiogenesis and facilitate extravascular invasion and immune escape, eventually leading to tumor progression and metastasis (28, 29). First, TAMs can promote tumor angiogenesis (29). Emerging studies have found that the amount of TAMs in the tissues of various tumors (such as breast cancer, lung cancer, glioma, gastric cancer, *et al.*) is positively correlated with the number and density of tumor blood vessels (20, 30). Various pro-angiogenic factors, such as vascular endothelial growth factor (VEGF), fibroblast growth factor (FGF) and matrix metallopeptidase 9 (MMP-9), are secreted to participate in the process of tumor angiogenesis (31). Second, TAMs can also mediate immunosuppression *via* interaction with various immune effector cells. It is currently reported that TAMs express the ligand receptors of programmed death 1 (PD-1) and cytotoxic T lymphocyte-associated antigen-4 (CTLA-4), which inhibit the activation of T cells. Studies have found that TAMs can produce not only immunosuppressive cytokines (IL-10 and transforming growth factor-*β* (TGF-*β*)), but also chemokines such as CCL5, CCL20, and CCL22 that recruit regulatory T cells into tumor tissues (20, 29). Third, TAMs support invasion and metastasis of tumor cells by increasing vascular extravasation, promoting survival and growth of metastatic cells, and suppressing effector T cells (20, 32). Ultimately pre-metastatic niche was established at distant sites in specific metastatic organs with the aid of macrophages. In the later sections, existing studies on the roles of macrophages in osteosarcoma will be further discussed.

## Macrophages and Osteosarcoma

### Macrophage Phenotypes in Osteosarcoma

Macrophages are one of the crucial immune components in the osteosarcoma niche. As described above, macrophages demonstrate a broad spectrum of activation status. Researchers mostly resort to the markers of the polarized extremes or a variety of cellular deconvolution methodologies to depict the heterogeneity of macrophages in osteosarcoma. A comprehensive study described that CD14^+^/CD68^+^ TAMs represent the main infiltrating immune cell types in bone sarcomas, including osteosarcoma (33). Similarly, an infiltration landscape of immune cells using the CIBERSORT algorithm showed a high ratio of M0 and M2 macrophages in osteosarcoma tissues in the TARGET cohort (34). Gene expression analysis and CD209 staining also confirmed the enrichment of M2 macrophages in human osteosarcoma tissues (35).

The progression and metastasis of osteosarcoma may induce an imbalance of macrophage subtype populations (36). It was reported that M2-like macrophage marker molecules, including CD206, Arg-1, and Ym-1, were significantly upregulated in the osteosarcoma tissues compared with non-tumor tissues (37). Another independent study also showed higher frequencies of CD163^+^ macrophages in tumor-infiltrating cells from resected tumors than in peripheral blood immune cells (38).

Notably, the infiltration of macrophage is also different in metastatic osteosarcoma. For instance, Han’s group observed that CD68 was significantly higher in osteosarcoma tissues of patients with detectable metastasis than patients without metastasis (39). Furthermore, the level of CD68 was also upregulated in human lung metastases than corresponding primary osteosarcoma lesions, while CD163, a biomarker of M2 macrophage, showed no significant difference (39). Additionally, Dumars et al. revealed a higher infiltration of the INOS^+^ M1 subtype in osteosarcoma tumors of non-metastatic patients (40).

Accordingly, some previous preclinical studies have come to the same conclusions. In a mouse model of human osteosarcoma implantation, macrophages were recruited into the tumor tissue and polarized into the M2 subset (41). Furthermore, it was found that a large number of F4/80^+^ cells were infiltrated into the metastatic pulmonary tissue (39) and M2-type (CD206^+^MHC-II^−^) macrophages were increased in the metastatic mouse lung tissue, but M1 (CD206^−^MHC-II^+^) remains unchanged (42). The levels of infiltrated M1 or M2 may vary in the primary and metastatic lesions (literature summarized in **Table 1**), suggesting their different role in the development of osteosarcoma.

**Table 1:** Macrophage phenotypes and their relation to clinical prognosis in osteosarcoma.

Species	Detection Methods	Markers of Phenotypes	Different Phenotypes of Infiltrating Macrophages	Prognosis Impact	Ref.
Human	Microarray and IHC analysis	Pan-marker: CD14; M1: HLA-DR*α*; M2: CD163	Higher CD14 expression in the non-metastasis group	Higher CD14^+^ macrophages correlated with metastasis suppression and better OS while M1or M2 not significant	(43)
Human	CIBERSORT algorithm	Not mentioned	M0 (0.23 ± 0.1) and M2 (0.24 ± 0.13) fraction of infiltrating immune cells	Higher M1 and M2 macrophages with better OS	(34)
Human	CIBERSORT algorithm	Not mentioned	Not mentioned	Higher M0 and lower M2 macrophages with better prognosis	(44)
Human	IHC analysis	M2:CD163; Pan-marker: CD68	High CD163 staining rate (43.8%) and high CD68 staining rate (23.4%)	Higher CD163 macrophages with better OS and MPFS	(45)
Human	IHC analysis	Pan-marker: CD68; M1: iNOS	Higher INOS^+^ macrophages in primary tumor tissues of patients of non-metastasis group	Higher CD68+ macrophages with Better OS	(40)
Human	IHC analysis	Pan-marker: CD68	Not mentioned	Higher CD68^+^ macrophage with poorer five year-EFS	(46)
Human	IHC analysis	Pan-marker: CD68; M2:CCL18	Higher CD68 in lung metastasis than primary osteosarcoma tissues	Higher CCL18^+^CD68^+^ macrophages with poorer prognosis	(47)
Human	IHC analysis	Pan-marker: CD14, CD68	ratio of CD14^+^/CD68^+^ TAMs relative to CD45^+^ cell (6–25%)	Not mentioned	(33)
Human	IHC analysis	M2: CD209	CD209 positive staining rate (78.57%)	Not mentioned	(35)
	Expression analysis of GEO data	M2: CD163, MRC1 and CCR2	higher gene expression levels of CD163, MRC1 and CCR2 in tumor	Not mentioned	
Human	IHC, PCR and WB analysis	M2: CD206, Arg-1 and Ym-1	Upregulation of CD206, Arg-1 and Ym-1 in osteosarcoma tissue than adjacent non-tumor tissue	Not mentioned	(37)
Human	FACS analysis	Pan-marker: CD14; M2:CD163	Higher CD14^+^/CD163^+^ macrophages in tumors than peripheral blood	Not mentioned	(38)
Human	IHC analysis	Pan-marker: CD68; M1: iNOS; M2: CD163	Higher CD68^+^ macrophages in primary tumor tissues of patients with metastasis	Not mentioned	(39)
			Higher CD68, lower iNOS and unchanged CD163 in metastasis than corresponding primary osteosarcoma tissues	Not mentioned	
NOD/SCID mice	FACS analysis	Pan-marker: F4/80; M2: CD163	Upregulation of CD163^+^/F4/80^+^ in 3 weeks after tumor implantation	Not mentioned	(41)
BALB/c nude mice	IHC analysis	Pan-marker: F4/80	Higher F4/80^+^ cells in lung metastases than corresponding primary osteosarcoma tissues	Not mentioned	(39)
BALB/c mice	FACS analysis	M1: MHC-II; M2: CD206	Higher CD206^+^/MHC-II^−^ macrophages and unchanged CD206^−^/MHC-II^+^ in metastatic mouse lung tissue than control lung tissue	Not mentioned	(42)

IHC, Immunohistochemistry; FACS, Fluorescence-activated Cell Sorting; WB, Western Blot; PCR, Polymerase Chain Reaction; TARGET, Therapeutically Applicable Research to Generate Effective Treatments; GEO, Gene Expression Omnibus.

### Relationship Between Macrophage Phenotypes and Clinical Prognosis

Macrophages have diverse functions and show plasticity in response to microenvironments. Mounting evidence suggests that macrophages facilitate tumor progression and metastasis (29, 48). In contrast to the tumor-supporting role for TAMs in many other tumor types, higher numbers of infiltrating TAMs correlated with better survival in osteosarcoma.

Several studies confirmed that the infiltration of macrophages, regardless of their polarization phenotype, exhibited positive clinical outcomes in osteosarcoma patients. Buddin et al. proved that TAMs defined as CD14-expressed cells were associated with metastasis suppression and better overall survival in high-grade osteosarcoma patients (43). A study based on the RNA-seq data and CIBERSORT algorithm analysis showed that higher M1 and M2 macrophages were associated with improved overall survival in prognosis (34). Consistent with this study, another bioinformatics analysis using different clinical dataset also observed that M0 macrophages were correlated with good prognosis (44). Similarly, Gomez-Brouchet et al. reported that upregulated CD163 TAM was significantly related to better overall survival and more prolonged metastasis progression-free survival (MPFS), and a similar trend was also observed for patients with higher levels of CD68-positive cell though not significant (45).

However, the relationship between CD68 positive macrophages (used to represent pan-macrophages) and the prognosis was controversial. Dumars et al. found that CD68^+^ TAM infiltration was positively correlated with better overall survival (40). In contrast, increased CD68^+^ macrophages in patients were reported to have poorer five-year-event free survival by Koirala et al. (46). Meanwhile, some studies also determined that the presence of M2-like macrophage showed opposite effects. Su et al. observed that CCL18/CD68 double-positive macrophages were significantly correlated with lung metastasis and worse prognosis in osteosarcoma patients (47). Intriguingly, Yang et al. also reported that M2 macrophages were correlated with poor prognosis in osteosarcoma patients, as seen in other types of tumors (44).

These discrepancies may be due to multiple factors, such as the different treatments before surgery or diverse experimental methods and details. For instance, Han et al. (39) adopted the surgically resected specimens after chemotherapy, while Gomez et al. (45) performed tissue analysis on the diagnostic biopsies from osteosarcoma patients without chemotherapy. Some studies included patients from public databases like the TARGET cohort (34) or GEO dataset (44), who varied largely in age, gender, tumor stage, tumor location, histologic grading, and metastatic status, resulting in unconformity in observing evidence (34). Meanwhile, the lack of consistency in macrophage markers used, as well as the lack of specificity of the selection of the current markers, may have contributed to the inconsistent results. Different markers were applied to serve as a pan-macrophage marker such as CD68 and CD14 in different studies, as described above. However, CD68 may be expressed on other non-myeloid origin cells, such as granulocytes, dendritic cells (49, 50). Apart from monocytes and macrophages, neutrophils and dendritic cells are positive for the marker CD14 (46). Most studies used CD163 as an M2-type TAM marker, yet CD163 expression may also be found in dendritic cells (51, 52). Notably, the activation status may also confuse the results. For example, Arginase-1 can be upregulated upon M1 (LPS) stimulation (17).

These studies suggested that the relationship between macrophage phenotypes and clinical prognosis in osteosarcoma was more complex than previously thought (literature summarized in **Table 1**). Based on the available evidence, we can conclude that macrophages are associated with the prognosis of osteosarcoma, whereas the adequate and homogeneous phenotypic characterization of macrophage subpopulations is lacking. These studies indicate macrophages exert distinct effects in osteosarcoma, though the specific subsets are still unclear and need comprehensive and thorough investigations. Moreover, osteosarcoma disease progression causes dynamic regulation of macrophage activation and reversion; thus, individual markers may not accurately evaluate the multifaceted and complex nature of the macrophage population (32). Harnessing new strategies, such as cell-fate mapping, single-cell sequencing, multicolor immunoﬂuorescence, and macrophage lineages targeting, may uncover the full spectrum of macrophage activation and give a landscape of the osteosarcoma tissue. Technical standardization and validation in a large scale of a clinical cohort with similar treatment and comparable clinical stages are required before the use of M1/M2 markers (53).

### The Role of Macrophages in Osteosarcoma

#### Inflammation Modulation

The inflammatory microenvironment is now recognized as an essential factor contributing to carcinogenesis, tumor metastasis, and treatment resistance (54, 55). However, inflammation in the microenvironment of osteosarcoma was recognized to have anti-tumor effects. It has been reported that post-operative infection was associated with improved survival in osteosarcoma patients (56, 57). Coley’s Toxins, which contained heat-killed bacteria or bacterial products, were utilized to treat bone sarcomas in the late 19th century (58, 59). Similarly, muramyl tripeptide, a synthetic derivative of the bacteria cell wall, was shown to play a positive role in treating osteosarcoma by activation of macrophage (58, 60). Therefore, macrophages, as the primary inflammatory cells stimulated by infection, may contribute to anti-tumor immunity (61).

Inflammation may enhance anti-tumor effects by increasing the level of infiltrating macrophages and secreting cytokine. A study based on chronic bacterial osteomyelitis mice model demonstrated that infection increased the number of TAMs and inhibited the growth of tumors in mice *via* regulating innate immune response elicited by macrophages. Moreover, the depletion of macrophages reversed these anti-tumor responses (62). Besides, infection upregulates the cytokine secretion of inflammatory macrophages, including tumor necrosis factor-α (TNF-α) and interferon-*γ* (IFN-*γ*), and reactivate the immune system towards anti-tumor response to attenuate immunosuppression induced by osteosarcoma (63). Although these models cannot precisely mimic the local inflammatory microenvironment of osteosarcoma, these preclinical researches, together with the above clinical data, provide a new understanding of the role of macrophages and the inflammatory response in osteosarcoma.

#### Involvement in Chemotherapy Resistance

Over the past 30 years, the application of adjuvant and neoadjuvant chemotherapy has significantly improved the 5-year survival rate to 60–70% for patients with osteosarcoma (3, 64). Despite treatment with chemotherapy, the 5-year event-free survival (EFS) in patients with recurrent osteosarcoma was 15–20%, and it seems unchanged over the years (3, 65).

**Figure 1:**
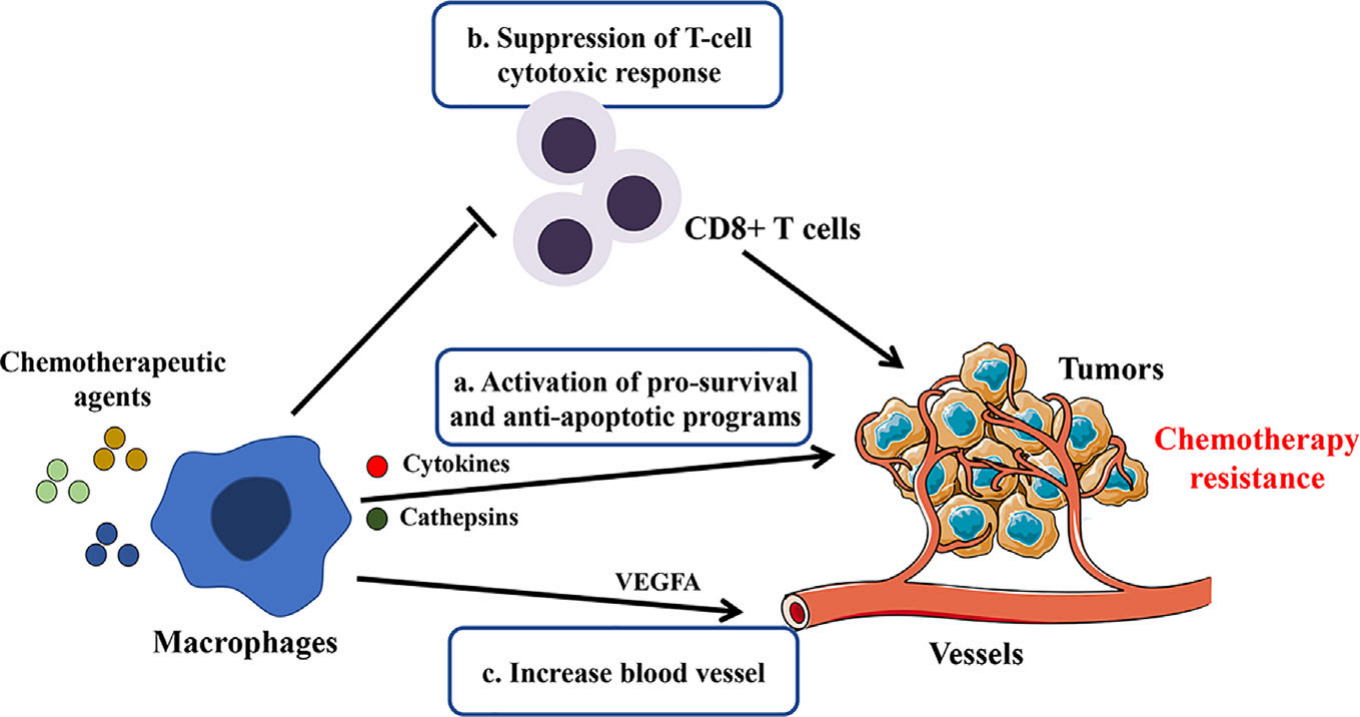
Potential mechanisms involved in macrophage-mediated resistance to chemotherapy. By secreting cytokines, growth factors, cathepsins, and exosomes or direct contact with tumor cells, TAMs blunt chemotherapeutic drugs’ efficacy by the following mechanisms: a. sustaining tumor cell survival; b. promoting immunosuppression; c. inducing tumor re-vascularization.

Although the mechanisms are uncertain, scholars have found that macrophages are closely linked to tumor resistance to chemotherapy (50). Chemotherapeutic drugs can inhibit tumorigenesis by blocking proliferation or promoting apoptosis of tumor cells while they induce tissue damage that inevitably activates macrophages’ tissue repair activities, resulting in pro-tumoral effects and drug resistance (66, 67). TAMs have been shown to hamper chemotherapy-induced anti-tumor responses in different ways, as illustrated in **Figure 1**. First, TAMs can sustain cell survival by secreting cytokines, growth factors, and exosomes (68–70). Those factors may contribute to the activation of anti-apoptotic programs and regulation of CSC activities (71). It was also found that macrophages protected against Taxol-induced tumor cell death partially by expressing cathepsins B and S (72). Second, immunosuppression induced by macrophages is also associated with chemotherapy tolerance of the tumors. DeNardo et al. found that inhibition of macrophage by CSF1R antagonists improved the survival of mammary tumor-bearing mice to paclitaxel by CD8^+^ T-cell-dependent mechanisms (73). Further, Ruffell et al. confirmed that IL-10 secreted by M2-type macrophages inhibits the expression of IL-12 by dendritic cells, thereby blocking the response of CD8^+^ T cells (74). Third, macrophages may also affect the vascularization and indirectly regulate the tumor sensitivity to chemotherapy (75, 76). VEGF-A clearance in macrophages led to normalized vascular growth and enhanced the sensitivity of Lewis Lung Carcinoma tumors to cytotoxic drugs like cyclophosphamide and cisplatin (77).

Furthermore, the roles of macrophages in chemo-resistance were evidenced by macrophage-targeting therapies (78). For instance, Lu et al. reported that the depletion of TAMs by CSF-1R inhibitors significantly improved the effects of docetaxel in a murine epithelial ovarian cancer model (79). Along the same lines, live imaging has demonstrated that the treatment with doxorubicin or cisplatin is improved in mice lacking CCR2^+^ TAMs (80). In the light of different cytotoxic agents and types of tumor, mechanisms accounting for TAM induced chemo-resistance need further investigation.

Several studies have confirmed this association between macrophages and chemo-resistance of osteosarcoma cells. Infiltrating CD68^+^ cells were higher in tumor tissues of osteosarcoma patients who were poorly reactive to neoadjuvant chemotherapy. Moreover, after treatment with chemotherapy medications, macrophages secreted IL-1β, which could activate downstream cancer signaling pathways and reduce the sensitivity of osteosarcoma to chemotherapeutic drugs. Moreover, blockage of the receptor of IL-1β restored the drug effects (81). Similarly, exosomes released by macrophages promoted proliferation, invasion and drug-resistance of osteosarcoma cells *via* the activation of AKT signaling, which has been widely recognized as a critical pathway mediated tumor progression (82). Those studies revealed that the secretome of macrophages might play a significant role in drug-resistance in osteosarcoma progression as in other tumors. Targeting macrophage provides potential strategies for improving the efficacy of neoadjuvant chemotherapy for osteosarcoma.

#### Involvement in Metastasis

It has been discovered that the number and varied polarization status of infiltrating macrophages were strongly correlated to the prognosis of osteosarcoma patients, as described above (39, 40, 43). Though previous studies came to inconsistent conclusions, macrophages were shown to enhance the metastatic process in osteosarcoma. Maloney et al. demonstrated that macrophage promoted the invasion of osteosarcoma cells and contributed to pulmonary metastasis in the animal model (42). Macrophages within the metastatic lung niche were altered to pro-tumor M2(MHC-II^−^/CD206^+^) phenotype and enhanced metastatic progression after the removal of the primary osteosarcoma tumor (83).

TAMs facilitate metastatic processes of osteosarcoma *via* several mechanisms. Su et al. found that CCL18 predominantly secreted by M2-type TAMs promotes proliferation and metastasis of osteosarcoma. Moreover, these effects were attributed to the upregulation of the lncRNA UCA1/Wnt/β-catenin pathway that mediated the tumor-promoting role in different types of tumors (47). Zhou et al. reported that M2 macrophages promoted the metastasis of osteosarcoma through secretion of matrix metalloproteinase 12 (MMP-12), which have been recognized as a metastasis-related factor and participate in degrading extracellular matrix (84). TAMs facilitated the expression of cyclooxygenase 2 (COX-2) of osteosarcoma cells and activate the COX-2/STAT3 axis and epithelial-mesenchymal transition (EMT) to promote osteosarcoma invasion and lung metastasis. Furthermore, blocking STAT3 or COX-2 could prevent the promoting-tumor effects of TAMs (39).

From clinical observations and preclinical studies, we can infer that TAMs, particularly M2-type macrophages, play a vital role in osteosarcoma invasion and metastasis.

#### Crosstalk Within the Microenvironment

In tumor microenvironments, the interaction between macrophages and other cells in osteosarcoma TME participates in the disease progress of osteosarcoma. As a part of their survival strategies, tumor cells often resort to cunning mechanisms to manipulate the macrophages and create an immunosuppressive, tumor-promoting microenvironment (85). Some studies demonstrated that osteosarcoma cells enhanced the recruitment of macrophages by secretion of cytokine. The increasing chemokine ligand 5 (CCL5) production by human osteosarcoma cells was reported to promote macrophages recruitment (86). Monocyte chemoattractant protein-1 (MCP-1, also called CCL2) expressed by osteosarcoma participated in the regulation of macrophage recruitment and infiltration *via* the MCP-1/CCR2 axis (87). IL-34 was released by osteosarcoma cells and promoted the recruitment of M2-TAMs into the tumor tissue, thus promote tumor growth and metastasis (22).

Interestingly, metastatic osteosarcoma cells display a more malignant phenotype *via* exosomal communication with macrophages. These exosomes significantly increased M2 macrophage-related cytokines such as IL10 and transforming growth factor-beta 2 (TGFB2), and modulate macrophages to a tumor-promoting M2 phenotype. This conversion contributed to the inhibition of macrophage-mediated tumoricidal functions like decreased phagocytosis, efferocytosis and direct tumor cell killing effects (88).

Macrophages may also influence the function of T cells. Han et al. revealed that the presence of M2-type (CD163^+^) macrophages was correlated with the frequency of TIM-3^+^ PD-1^+^T cells representing the exhausted and immunosuppressive T cell subset. And these macrophages contributed to the impairment of T cell proliferation and production of pro-inflammatory cytokine and hence aggravated immunosuppression. Additionally, selective depletion of CD163(+) macrophages revive T cell function (38).

The initiation and progression of osteosarcoma result from a complex interaction of the integral microenvironment constructed by several types of cells and matrix (89). Further intensive studies would provide a better understanding of the interplay between macrophages and other cells.

### Macrophage Targeting Therapeutics in Osteosarcoma

#### Macrophage Depletion and Recruitment Targeting

One of the macrophage-targeting treatments is to reduce the number of infiltrating TAMs. The main therapeutic strategies include direct depletion of macrophages and reduction of monocyte/macrophage recruitment. After being engulfed, clodronate liposomes can eliminate macrophages *via* the induction of apoptosis (90). Regarding the preclinical studies of osteosarcoma, researchers have confirmed that the clodronate liposomes treated mice demonstrated reduced lung metastasis of osteosarcoma (42, 84) and decreased tumor growth (41).

Several cytokines and chemokines were confirmed to be involved in the recruitment of macrophages (91). For example, CCL2 is a member of the C-C type chemokine family secreted by tumor cells or TAMs to promote TAMs recruitment (92, 93). It has been reported that Bindarit, a specific inhibitor of CCL2, efficiently reduced the infiltration of macrophages and inhibited the growth of the osteosarcoma tumor (87).

Due to the pro-tumor effects of TAMs in osteosarcoma, decreasing TAMs present in the tumor by macrophage-eliminating agents or some specific inhibitors may achieve an excellent therapeutic effect.

#### Macrophage-Related Immune Checkpoint: CD47/SIRP*α*

The regulation of macrophages affects tumor development, and the application of immunomodulatory therapy to enhance anti-tumor effects is getting more and more attention (94). Specific blocking of receptor-ligand binding between macrophages and the tumor cells can enhance macrophage phagocytosis and anti-tumor activity, thus appears to be a promising strategy for cancer therapy (95, 96). CD47 is recognized as a ‘don’t eat me’ signal, which binds to signal regulatory protein *α* (SIRP*α*) in the surface of macrophages resulting in the escape of phagocytosis and cell death (97, 98). As previously reported, CD47 is expressed in a variety of solid tumors and hematologic tumors (99). It was reported that CD47 was overexpressed in human osteosarcoma samples of different types than normal bone tissue or osteoma samples (100, 101). Similar to the preclinical studies in other tumors, CD47 can represent a useful therapeutic target in osteosarcoma. It has been confirmed that CD47 blockade by specific antibodies promotes the phagocytic effects of macrophages on osteosarcoma cells (101, 102). CD47 mAb treatment combined with chemotherapy increased the number of macrophages and further enhanced their phagocytic capabilities in osteosarcoma, thus produced a better outcome in the osteosarcoma-bearing mice model (103). Another study showed that SIRP-*α* knockout macrophages boost phagocytosis of osteosarcoma tumor cells (104).

Based on the preclinical evidence, several clinical trials are performed with CD47/SIRPα blocking using mAbs or Fc fusion proteins either alone or in combination with other therapies to treat different tumors (105–107). Those clinical trials are ongoing on multiple hematologic malignancies, including acute myeloid leukemia and myelodysplastic syndrome, and some advanced solid tumors such as liver cancer, non-small cell lung cancer, ovarian cancer, et al. (http://www.clinicaltrials.gov). However, there are no registered clinical trials on osteosarcoma patients so far. Compared with the first generation of CD47 targeting drugs terminated in trials due to their considerable side effects, the newly developed antibodies now being tested exhibit minimal binding to CD47-expressing red blood cells, minimizing their potential toxicity related to hemolytic anemia (108). For instance, Hu5F9-G4 (5F9), an anti-CD47 monoclonal antibody, was well tolerated in patients with advanced cancers and generated objective responses in the phase I trial (NCT02216409) (109). Moreover, 5F9 combined with rituximab (a CD20 antibody that targets B cells) exhibited promising activity in the treatment of B-cell lymphomas (NCT02953509) (110).

Although there are limited studies on anti-CD47/SIRP*α* therapy in osteosarcoma, these suggested strategies targeting CD47/SIRP-*α* that turn the ‘don’t eat me’ signal off may be an efficient therapy in osteosarcoma.

#### L-MTP-PE: Macrophages Activator

Mifamurtide, as an immunostimulatory agent, is one of the most critical advances in macrophage targeted therapy of osteosarcoma (111). Liposomal muramyl tripeptide phosphatidyl ethanolamine (L-MTP-PE or mifamurtide) is derived from muramyl dipeptide (MDP), which is a component of bacterial cell walls (112). L-MTP-PE may serve as an immunomodulator to activate macrophages and monocytes, and potentiate tumoricidal activity, causing the suppression of tumor growth and metastasis. L-MTP-PE is far more efficient than MDP in activating macrophages (113). Macrophages activated with L-MTP-PE resulted in tumor cell destruction without leading to resistance of tumor cells (114). Mifamurtide can upregulate the markers of M1 and M2 thus modulate macrophages into an M1/M2 intermediate phenotype, which achieved a dual role in anti-tumor and immunomodulatory functions (115).

Induction of soluble cytokines such as TNF-α and IL1-β plays a role in the mechanism of action of L-MTP-PE on macrophages (116). Moreover, these cytokines may contribute to the functions of other immune cells (58). L-MTP-PE administration stimulated the production of cytokines such as TNF-α and IL-6 in patients with osteosarcoma (60).

Several studies also determined the efficacy of combination treatment with L-MTP-PE. Pahl et al. reported that in combination with interferon-*γ*, L-MTP-PE activated macrophages to inhibit the growth of osteosarcoma cells (116). L-MTP-PE alone or in combination with doxorubicin (DOX) was found to enhance the cytotoxic activity of macrophages against osteosarcoma in a canine model (117). In a clinical trial of patients with osteosarcoma, MTP combined with chemotherapy resulted in better clinical outcomes (118), including improved overall survival and a trend toward better event-free survival (119).

Existing evidence proves that L-MTP-PE acts as a potent activator of the immune response of macrophages and can be used in anti-osteosarcoma therapy.

#### Regulation of Macrophage Polarization

One of the critical characteristics of macrophages is their plasticity; thus, macrophages can respond to various stimuli in the TME, leading to a broad spectrum of activation phenotypes. As described above, the M2-like polarization of macrophages contributes to the pulmonary metastasis of osteosarcoma. Therefore, re-educating TAMs from immunosuppressive and pro-tumoral macrophages to the anti-tumor phenotype is a promising tumor treatment strategy, compared to depletion therapies targeting all macrophages.

Several approaches have been attempted to reprogram the TAMs, include cytokines, Toll-like receptors (TLRs) agonists, monoclonal antibodies (120). Many factors are known to repolarize TAMs towards an M1-like phenotype, such as IFN-γ, IL-12, leading to the activation of the STAT signaling pathway (121). TLRs are essential pathogen recognition receptors expressed by antigen-presenting cells, including macrophages. TLRs agonists induce the conversion of M2 to M1 phenotype to elicit anti-tumor effects (122, 123). For instance, Vidyarthi et al. reported the administration of TLR-3 ligand [poly (I: C)] in the murine colon tumor skewed the M2-macrophages to M1-phenotype and regressed the tumor growth in the IFN-αβ signaling pathway-dependent manner (124). In addition to cytokines and TLR agonists, antibodies like anti-CSF1 and anti-CD40 were also applied to skew TAM polarization (120, 121).

In osteosarcoma, several drugs were elucidated to repolarize the macrophages and showed promising results. M1-like macrophages activated by LPS plus IFN-*γ* showed suppression on osteosarcoma cell growth, and those effects were mediated by soluble factors secreted by macrophage in a TNF-α/IL-1-independent manner (116). All-trans retinoic acid (ATRA) inhibited osteosarcoma invasion and metastasis by suppressing M2 polarization and secretion of MMP12 (84). Furthermore, this research team reported that ATRA could prevent M2-type macrophage-mediated enhancement of osteosarcoma initiation and tumor cell stemness (35). Metformin, which was previously reported to elicit anti-tumor and anti-angiogenic effects by repolarization of macrophages, also contributes to osteosarcoma’s growth inhibition *via* redirecting the metabolism polarization of macrophages (125, 126).

Intriguingly, gefitinib, an epidermal growth factor receptor (EGFR) inhibitor, altered pulmonary macrophage phenotype to block osteosarcoma invasion and reduce metastatic burden *via* inhibition of macrophage receptor-interacting protein kinase 2 (RIPK2) (42). Moreover, gefitinib altered macrophage phenotype and relieved surgery-accelerated metastasis and prolonged overall survival in mice model (83).

Fujiwara et al. identified a series of compounds screened from natural substances, namely, Onionin A1 (derived from Allium Sulfides) (127), epimedokoreanin B (a compound from Epimedii Herba) (127), and corosolic acid (CA)/oleanolic acid (OA) (both are triterpenoid compounds) (128). Those compounds possessed an inhibitory effect on the M2-macrophage polarization by suppressing STAT3 activation and preventing osteosarcoma progression and metastasis in osteosarcoma mice model.

Another research team also concentrated on the development of M2-type macrophage inhibitors/modulators, including wogonin (isolated from Scutellaria baicalensis roots) (129), dihydroxycoumarins (esculetin or fraxetin) (130), xanthoangelol and 4-hydroxyderricin (derived from Angelica keiskei roots) (131), resveratrol (132) and synthetic hydroxystilbenes (133). They examined that these substances effectively inhibit osteosarcoma growth and metastasis *via* suppression activation and differentiation of M2 macrophages.

Therefore, targeting the regulation of TAM polarization is a potential strategy for anti-osteosarcoma therapy.

#### PD-1/PD-L1 Inhibitors

PD-1/PD-L1 inhibitors as a means of tumor immunotherapeutics have been successfully applied clinically in treating a variety of tumors (134, 135). Their interaction with macrophages in the tumor microenvironment has also attracted increasing attention (136).

Several studies have revealed that PD-L1 expression was observed in primary and metastatic tumors of osteosarcoma patients (46, 137). PD-L1 positive tumors compared to PD-L1 negative tumors was significantly correlated with the presence of macrophages (137), particularly CD68^+^ cells (46, 138), implicating the potential role of macrophages in the anti-PD1/PD-L1 treatment. TAMs also expressed PD-1 to participate in immune escape and inhibit phagocytosis and anti-tumor immunity (139). Moreover, the infiltrating macrophages were largely PD-L1 positive (up to 45%) in osteosarcoma (137). This evidence suggests that targeting tumor-associated macrophages may represent an additional means to improve PD1/PD-L1 blockage therapy.

Additionally, some studies showed the effects of anti-PD1/PD-L1 therapies by acting on macrophages. Anti-PD1 treatment decreased lung metastases of osteosarcoma through activating CD86^+^ M1 and reduced CD16^+^ M2 macrophages. Moreover, it was confirmed that macrophage depletion significantly compromised anti-PD1 efficacy (140). Similarly, it has been reported that anti-PD-L1 treatment blocks the PD-L1 signaling pathway, promoting macrophage proliferation and activation, leading to pro-inflammatory macrophage phenotypes (141). In an osteosarcoma mice model, the PD-L1 inhibitor also promoted monocyte maturation and returned macrophage M1/M2 marker expression to nearly normal status (36).

These studies suggest a new theoretical application of anti-PD-1/PD-L1 antibodies alone or combination therapy to treat osteosarcoma.

## Conclusions

In summary, macrophages are associated with clinical prognosis and possess clinically applicable potential in osteosarcoma treatment. As described above, macrophages, predominantly M2-type TAMs, promote the osteosarcoma metastasis and exert pro-tumor effects. Biomarkers, such as CD163, CD209, CCL18, *et al*., have been correlated with tumor progression in preclinical models of osteosarcoma. Furthermore, based on the immunoscore combined with a series of macrophages markers (not a single indicator), an algorithm can be constructed to differentiate patients and support diagnosis and the corresponding treatments and prognosis. For instance, Gomez and his colleagues (45) proposed a systematic analysis of CD68, CD163, CD8, PD1 and PDL-1 expression performed in osteosarcoma biopsies to stratify patients regarding their respective TME and suggested a therapeutic strategy targeting macrophages and other immunological factors. Development and validation of a TAM-based immune signature will afford a valuable clinical decision-making tool to screen subpopulations that respond and benefit most from the current therapies.

The current studies demonstrate that macrophages are involved in the local inflammation modulation, invasion, metastasis, and chemotherapy resistance of osteosarcoma and further interacted with other cells in TME. However, the distinct TAM subtypes may differentially react to osteosarcoma disease. Selective targeting the TAMs (such as CD163(+) TAMs (38, 142)) rather than pan-depletion demonstrated improved T-cell cytotoxic function tumor regression. Such information might prompt researchers to define specific TAM signatures and subsets in human biopsies for effective TAM targeting therapies. In fact, specific TAM subset(s) features and signals continuously evolve along with the disease history, regulating either pro- or anti-tumor activity. As such, the complex roles and detailed mechanisms of macrophages in osteosarcoma still need further exploration.

Based on present studies, the phenotypes or polarization states of macrophages of osteosarcoma were not well recognized. These results might appear to be contradictory due to the inconsistent definitions of M1/M2 markers and different specimens. Notably, the already described multiple biological functions of TAMs engaged in different in many types of tumors suggested that such definitions are insufficient and limiting and can hardly represent the whole dynamic process of TAMs in the TME. A thorough characterization of macrophages based on pathophysiological function rather than merely preexisting nomenclature is also needed.

**Figure 2:**
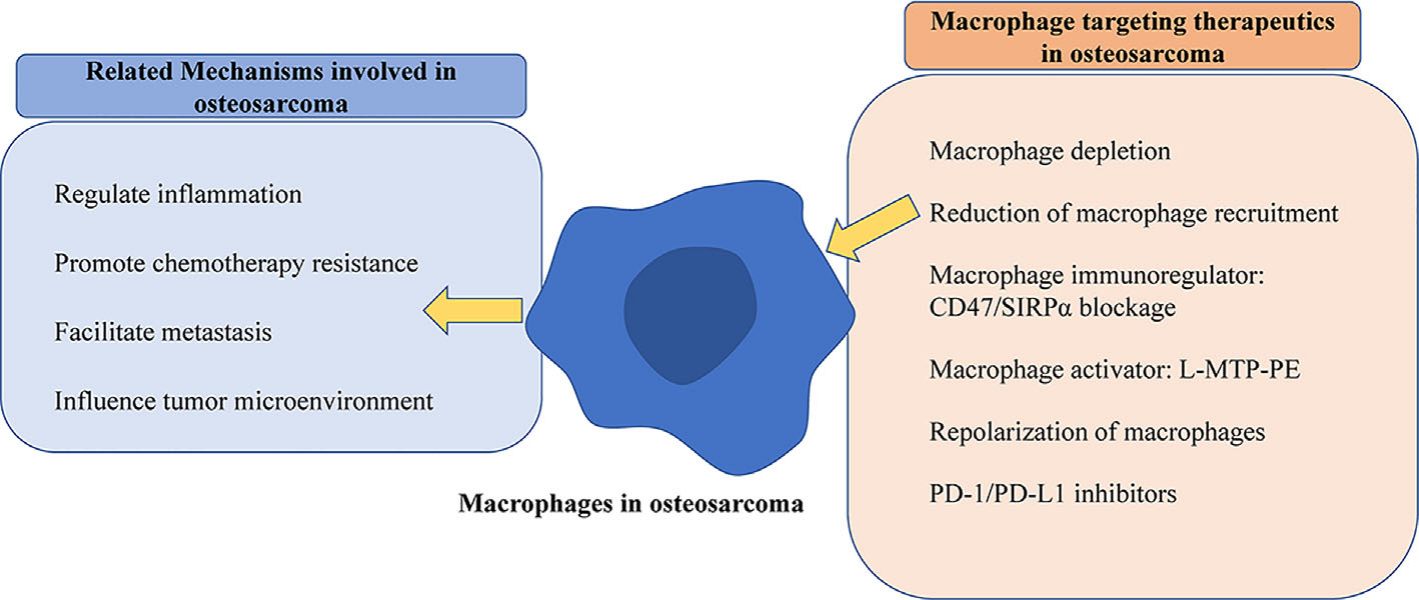
Schematic diagram of mechanisms of macrophages and targeting strategies in osteosarcoma. The left part exhibits that macrophages play various roles, and the right part shows validated treatments by macrophage targeting in osteosarcoma.

Recently researchers, encouraged by successes in treatments with immune checkpoint blocking in some other types of malignancies, made efforts to capitalize on advances by extending those regimens to osteosarcoma patients. However, osteosarcoma is characterized by relatively low immunogenicity, which may partly explain the low objective response to PD-1 Ab monotherapy treatment in the clinical trial (143, 144). A better understanding of macrophages allows the development of novel macrophage targets, and combines TAM-targeting approaches with other therapeutic approaches, which is of great significance to provoke immunotherapeutic responses in osteosarcoma patients. The primary clarified function and targeting therapeutics of macrophages in osteosarcoma were summarized by the schematic diagram shown in **Figure 2**.

## Author Contributions

Z-WL prepared the original draft of the manuscript. HX conceptualized, reviewed, and edited the manuscript, and supervised the study. P-PL, Z-XW, and C-YC revised and edited the manuscript. All authors contributed to the article and approved the submitted version.

## Funding

This research and the APC was funded by the National Natural Science Foundation of China (Grant Nos. 81670807, 81871822, 81702237, 81801395), the Excellent Young Scientist Award of National Natural Science Foundation of China (Grant No. 81522012), the Thousand Youth Talents Plan of China (Grant No. D1119003), the Medicine and Health Science and Technology Innovation Project of Chinese Academy of Medical Sciences (Grant No. 2019-RC-HL-024), the High Level Talent Gathering Project of Hunan Province (Grant Nos. 2017XK2039, 2018RS3029), and the Innovation Driven Project of Central South University (Grant Nos. 2016CX028, 2019CX014).

## Conflict of Interest

The authors declare that the research was conducted in the absence of any commercial or financial relationships that could be construed as a potential conflict of interest.
